# Effects of Silver Nanoparticles on Radish Sprouts: Root Growth Reduction and Modifications in the Nutritional Value

**DOI:** 10.3389/fpls.2016.00090

**Published:** 2016-02-16

**Authors:** Nubia Zuverza-Mena, Raul Armendariz, Jose R. Peralta-Videa, Jorge L. Gardea-Torresdey

**Affiliations:** ^1^Metallurgical and Materials Engineering Department, The University of Texas at El PasoEl Paso, TX, USA; ^2^Chemistry Department, The University of Texas at El PasoEl Paso, TX, USA; ^3^University of California Center for Environmental Implications of Nanotechnology – The University of Texas at El PasoEl Paso, TX, USA

**Keywords:** silver nanoparticles, radish, FTIR, elemental analysis, macromolecules

## Abstract

Reports indicate that silver nanoparticles (nAg) are toxic to vegetation, but little is known about their effects in crop plants. This study examines the impacts of nAg on the physiology and nutritional quality of radish (*Raphanus sativus*) sprouts. Seeds were germinated and grown for 5 days in nAg suspensions at 0, 125, 250, and 500 mg/L. Seed germination and seedling growth were evaluated with traditional methodologies; the uptake of Ag and nutrients was quantified by inductively coupled plasma-optical emission spectroscopy (ICP-OES) and changes in macromolecules were analyzed by infrared (IR) spectroscopy. None of the nAg concentrations reduced seed germination. However, the water content (% of the total weight) was reduced by 1.62, 1.65, and 2.54% with exposure to 125, 250, and 500 mg/L, respectively, compared with the control. At 500 mg/L, the root and shoot lengths were reduced by 47.7 and 40%, with respect to the control. The seedlings exposed to 500 mg/L had 901 ± 150 mg Ag/kg dry wt and significantly less Ca, Mg, B, Cu, Mn, and Zn, compared with the control. The infrared spectroscopy analysis showed changes in the bands corresponding to lipids (3000–2800 cm^-1^), proteins (1550–1530 cm^-1^), and structural components of plant cells such as lignin, pectin, and cellulose. These results suggest that nAg could significantly affect the growth, nutrient content and macromolecule conformation in radish sprouts, with unknown consequences for human health.

## Introduction

The “Nano-Era” started in the late 1990’s propelled by the worldwide increase in government investments into nanomaterials (NMs) and their applications. In the United States, the National Nanotechnology Initiative, created at the beginning of 2000, had coordinated the research and development of nanotechnology (Roco, 2003). Since then, a number of carbon-based and metal-based NMs have been produced and are currently being used in many fields. NMs are commonly referred to as small objects with one or more external dimensions in the size range 1–100 nm. At these dimensions, materials exhibit a distinctive behavior in comparison to larger particles of the same composition. Silver is an important component in the area of NMs, with over 450 metric tons of silver nanoparticles (nAg) produced in 2010 (Keller et al., 2013). In November 2014, fifty percent of the nano-enabled products on the “Project on Emerging Nanotechnologies” inventory contained nAg^1^. Among other goods, listed products included wound dressings with bactericidal effects that enhance healing, antibacterial door knobs and anti-odor socks; all articles containing nAg that provide antimicrobial properties (Kim et al., 2007). At some point, it is assumed that all the nanoparticles (NPs) in different items will end up in the soil, air or water (Keller et al., 2013). Even though some studies have reported the effects of nAg in the environment (Daniel et al., 2010; Beer et al., 2012), their toxicity on crop plants is not yet well understood. Previous investigations indicate that the effects of nAg on seed germination vary with NP characteristics and plant species. Kumari et al. (2009) reported that nAg decrease the mitotic index and cause multiple chromosomal breaks and cell disintegration in onion (*Allium cepa*). Stampoulis et al. (2009) found that nAg at 500 and 100 mg/L reduced plant biomass and transpiration in zucchini *(Cucurbita pepo*) by 57 and 41%, respectively. In another experiment, *C. pepo* sp. *ovifera* was exposed in hydroponics to 0, 100, and 500 mg nAg/L and corresponding bulk Ag (Ag powder). Results showed more negative effects in plants exposed to nAg than bulk Ag (Musante and White, 2012). It has also been observed that nAg reduced growth in mung bean (*Phaseolus radiatus*) and sorghum (*Sorghum bicolor*) cultivated in soil or agar-based medium (Lee et al., 2012). In addition, Pokhrel and Dubey (2013) found that coated nAg promoted histological changes in maize (*Zea mays* L.) by inducing elongation of root cells, and reduced root growth in cabbage (*Brassica oleracea* var. *capitata* L.) by 24%. In tomato (*Solanum lycopersicum*), nAg did not affect germination even at 5000 mg/L (Song et al., 2013). Thuesombat et al. (2014) reported that the toxicity of nAg in rice (*Oryza sativa* cv. KDML 105) increased directly with the particle size and concentration. A more recent report indicates that nAg at a concentration of 100 mg/L reduced germination in *Brassica nigra* (Amooaghaie et al., 2015). However, the effects of nAg on radish have not been reported yet. Radish (*Raphanus sativus* L.) sprouts are widely consumed worldwide due to their nutritional content and antioxidant properties (Xiao et al., 2014; Baenas et al., 2015). More than two decades ago, radish was proposed as a model plant for the study of environmental stresses, mainly atmospheric contaminants (Kostka-Rick and Manning, 1993). More recently, due to its short growing period, this plant has been considered as a model of edible roots for the study of the interaction of plants with soil contaminants (Létondor et al., 2015). A few reports have shown different responses of radish seedlings exposed to NMs. Ma et al. (2010) reported that nLa_2_O_3_, nGd_2_O_3_, and nYb_2_O_3_ at 2000 mg/L inhibited root elongation. However, Trujillo-Reyes et al. (2013) found that citric acid coated nCeO_2_, at 200 mg/L, increased root biomass and seedlings’ water content. In addition, Corral-Diaz et al. (2014) reported that nCeO_2_ at 250 mg/kg soil increased radish tubers’ antioxidant capacity. There is concern about the trophic transfer of NPs from edible plants into the food chain (Gardea-Torresdey et al., 2014). The present investigation addresses the effects of a nAg suspension, intended for human ingestion, in a terrestrial plant. In this study, radish seeds were exposed to different nAg concentrations from a commercially available nAg suspension to test its effects on radish sprouts. The marketed nAg product, at 500 mg/L per serving, is indicated as a dietary supplement for immune support^2^. Even though the environmental concentrations of nAg are lower than the amounts used for this study (Gottschalk et al., 2013), we chose 500 mg/L as the highest concentration for the experiment, assuming the worst case scenario for this product. The effects on seedlings’ development, nutrient uptake and changes of macromolecules were studied by using spectrophotometric analytical techniques.

## Materials and Methods

### Silver Nanoparticles and Radish Seeds

Silver nanoparticles (nAg) from Natural Path/Silver Wings (Nashville, TN, USA) came suspended in deionized water at 500 mg/L. According to the manufacturer, the majority of the nAg are 2 nm in size forming colloids in the range of 1-10 nm. The hydrodynamic size of the suspended particles in water and the zeta potential (ζ, the electrostatic charge between particles) was analyzed by dynamic light scattering (DLS) using a Malvern Zetasizer (Nano-ZS90, Malvern Instruments, UK). Radish (Champion variety) seeds were obtained from Del Norte Seeds and Feed (Vinton, TX, USA).

### Seed Germination

Thirty seeds were directly incubated without previous treatment in sterilized standard Petri dishes (10 cm diameter) over germination paper, modified from López-Moreno et al. (2010). Treatments consisted of nAg suspensions at 0 (control), 125, 250, and 500 mg/L; four replicates per treatment. The concentrations for the study were selected considering that the worst case scenario at which plants could be exposed is the commercially available product of nAg at 500 mg/L. Suspensions were prepared by diluting the stock suspension of 500 mg/L (as supplied by the vendor) with Millipore water (18 MΩ cm). We utilized Millipore water for the experiments because it has similar resistivity than deionized water (Yin et al., 2012). Aliquots of five milliliters of nAg suspension were administered to each Petri dish, except for control seeds that received five milliliters of Millipore water. The dishes containing the seeds were covered with aluminum foil for 3 days. Then, they were set into a growth chamber (Environmental Growth Chamber, Chagrin Falls, OH, USA), where seedlings grew for a total of 5 days before analysis. Environmental conditions inside the chamber were 25/20°C day/night temperature, 14/10 h light/dark photoperiod, 60 ± 3% relative humidity, and 340 μmol/m^2^s light intensity. The percent germination (%G), relative germination (%RG), and germination change (%GC) were calculated as per de la Rosa et al. (2011). The length of the roots and shoots was measured on 15 plants per replicate. Water content, fresh and dry weights (dry wt) were also determined on 15 plants per replicate.

### Elemental Analysis

At harvest, seedlings were washed with 0.01 M HNO_3_ and rinsed with Millipore water to remove the nAg adhered to tissues. After washing, seedlings were oven dried at 70°C for 72 h (Corral-Diaz et al., 2014). Dried samples were prepared for analysis, according to the EPA method 3051. Briefly, the seedlings were powdered using mortar and pestle and acid digested (0.1 g per sample) with a 1:4 ratio of HNO_3_:H_2_O_2_ in a microwave oven (MarsX, CEM Mathews, NC, USA). Digested samples were placed in 15 mL polypropylene centrifuge tubes and the final volume was adjusted to 15 mL. The digests were analyzed for macronutrients, micronutrients, and Ag content by using inductively coupled plasma-optical emission spectroscopy (ICP-OES, Perkin Elmer Optima 4300 DV, Shelton, CT, USA). For quality assurance/quality control, a standard of 0.5 mg/L from the calibration curve that contained Ag, micro and macro elements was analyzed every five samples.

### FT-IR Analysis

Changes in lipids, proteins, carbohydrates and other organic polymers (e.g., lignin) were studied by using Fourier transform infrared (FT-IR) spectroscopy. The sprouts were washed with 0.01 M HNO_3_ and rinsed with Millipore water, separated into roots, stems and leaves and oven dried at 70°C for at least 72 h. Samples of roots, stems, and leaves were analyzed by using a Perkin-Elmer, Spectrum 100 with a Universal Attenuated Total Reflectance (ATR) sampling accessory. Background correction was performed by acquiring a spectrum without sample. Powdered samples were placed on the spectrometer stand and spectra were recorded. The data was collected in a frequency range from 4000 to 600 cm^-1^ at a resolution of 1 cm^-1^ and three scans per reading. Results are averages of triplicate determinations, as similarly reported by Servin et al. (2013). The amide I peak is commonly taken as an internal standard to normalize biological samples’ spectra (Yu and Irudayaraj, 2005). Data was normalized at wavenumber 1650 cm^-1^ for roots, stems, and leaves. This allowed us to compare the spectra within the same plant tissue at different nAg concentrations.

### Statistical Analysis

Data was analyzed using the Statistical Package for the Social Sciences 20.0 (SPSS, Chicago, IL, USA). Variance was evaluated by one-way analysis of variance (one-way ANOVA) and the difference between treatment means was compared by Tukey’s honest significant difference (Tukey’s HSD) test at a *p*-value of 0.05, unless otherwise stated. Regression analysis was performed on growth data.

## Results

### Characterization and Effects of nAg on Seed Germination

The nAg suspended in Millipore water had (at the highest concentration) a hydrodynamic size of 77 ± 2.44 nm and a zeta potential (ζ) of -24.4 ± 12.6 mV. Results suggest that nAg may be aggregating given that the manufacturer specifications indicate a colloid size of 1–10 nm. Note that our recorded dimensions (77 ± 2.44 nm) include any layer that forms around the nAg due to interactions with the aqueous media, where the inorganic complex is suspended (Supplementary Figure S1).

Supplementary Figure S2 shows an overall view of the experimental setup, while Supplementary Figure S3 shows enlarged views of one Petri dish/treatment. The germination percent and changes in germination of radish seeds exposed to nAg are shown in **Table 1**. As seen in this table, at 125 mg/L nAg increased the germination by 3%, while at 250 and 500 mg/L reduced the germination by 3 and 6%, respectively. However, none of the treatments reached a statistical significant difference in comparison to the control.

**Table 1:** Germination percent (%G), relative germination percent (%RG) and germination change (GC%) of radish seeds after 5 days of incubation in nAg suspension at 0, 125, 250, and 500 mg/L.

	(Control) 0 mg/L	125 mg/L	250 mg/L	500 mg/L
%G	93 ± 1	96 ± 3	90 ± 4	88 ± 1
%RG	100 ± 1	103 ± 3	97 ± 4	94 ± 1
%GC	0 ± 1	3 ± 3	-3 ± 4	-6 ± 1

*Results are means of four replicates ±standard error*.

### Seedling Growth, Silver Uptake, Biomass Production, and Water Content

**FIGURE 1:**
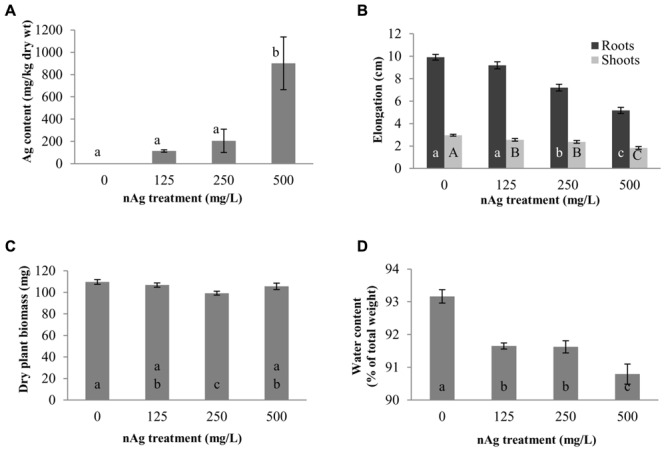
**Concentration of Ag, mg per kg of dry plant tissue (A), root and shoot length (B), dry biomass (C), and water content (D) in radish seedlings exposed for 5 days to nAg at 0 (control), 125, 250, and 500 mg/L.** Values are means of four replicates per treatment (15 plants each replicate) ± standard error. Different letters denote statistically significant differences according to the Tukey’s HSD test (*p* < 0.05). In **(B)**, small case letters are for roots and upper case letters for shoots.

**Figure 1** shows the root and shoot elongation, dry biomass production, water content, and Ag concentration in seedlings exposed to nAg at 0 (control), 125, 250, and 500 mg/L. Individual images of seedlings from the different treatments are shown in Supplementary Figure S4. As seen in **Figure 1A**, accumulation of Ag was concentration dependent, but there was no difference between 125 and 250 mg/L treatments (114 and 204 mg Ag/kg dry tissue, respectively). However, at 500 mg/L, Ag accumulation was significantly higher (900 mg Ag/kg dry tissue) compared to the other treatments (*p* ≤ 0.05).

The elongation of roots and shoots is shown in **Figure 1B**. As shown in this figure, there was a concentration-dependent reduction in root elongation (*r*^2^ = 0.9626) that reached statistical significance in seedlings exposed to 250 and 500 mg/L with respect to control. The percent reductions in the two treatments were 27.3 and 47.7%, respectively. In addition, the root length of seedlings exposed to 500 mg/L (5.2 cm) was statistically lower compared to the length of roots exposed to 250 mg/L (7.2 cm). **Figure 1B** also shows the shoot length of radish seedlings. Similar to root length, there was a concentration-dependent significant reduction (*r*^2^ = 0.9677) in shoot elongation. However, in the case of shoots, all nAg concentrations significantly reduced shoot elongation, although the reduction at 125 and 250 mg/L was statistically similar. At 500 mg/L nAg exposure, the reduction in shoot length reached 38%.

The effects of nAg on dry biomass are shown in **Figure 1C**. Plants exposed to 250 mg/L had less biomass, compared with control and the other treatments. The reduction was about 10%, compared with control, and about 7% compared with the other treatments.

Water content of the whole seedlings is shown in **Figure 1D**. As shown in this figure, the water content (expressed as % of the total seedlings’ weight) was reduced by 1.62, 1.65, and 2.54% with exposure to 125, 250, and 500 mg/L, respectively, compared with the control. In all cases the differences were statistically significant, compared with the control; however, there were no differences between the 125 and 250 mg/L treatments (*p* ≤ 0.05).

### Effects of nAg on Macro and Micronutrient Accumulation

**FIGURE 2:**
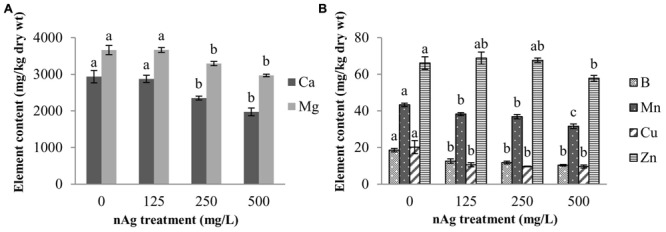
**Concentration of macroelements (A) and microelements (B) in radish seedlings germinated and grown for 5 days in nAg suspensions at 0 (control), 125, 250, and 500 mg/L.** Values are means of four replicates per treatment (15 plants each) ± standard error. Different letters denote statistically significant differences according to the Tukey’s HSD test (*p* < 0.05).

**Figure 2** shows the concentration of elements that were affected by nAg in the whole seedling. Amongst macroelements, only Ca and Mg were significantly reduced by the highest concentrations of nAg (**Figure 2A**). Calcium was reduced by 20 and 33% and Mg by 10 and 19% at 250 and 500 mg/L, respectively, compared with control.

With regard to micronutrient absorption, none of the nAg concentrations impaired the absorption of Fe, Ni, and Mo. However, all treatments significantly reduced (*p* ≤ 0.05) absorption of B, Mn, and Cu, while Zn was only reduced at 500 mg/L, compared with control (**Figure 2B**). Percent reductions at 125, 250, and 500 mg/L were 32, 36.8, and 44.6% for B; 11.6, 14.8, and 26.9% for Mn, and 47.5, 52.5, and 52.5% for Cu, respectively. Zinc was reduced by 12.6% at the highest concentration treatment.

### FT-IR Analysis of Roots, Stems, and Leaves

**FIGURE 3:**
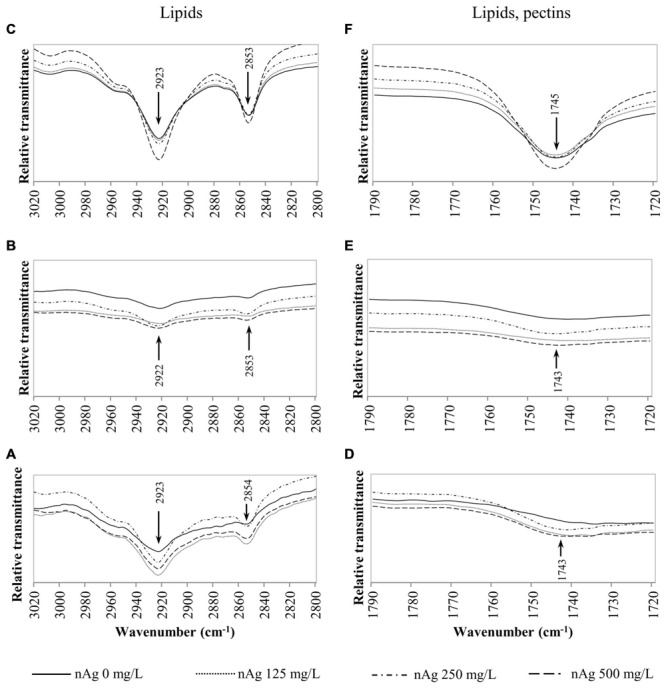
**Overlap ATR-FTIR spectra of radish sprouts exposed to nAg at 0, 125, 250, and 500 mg/L.** Spectral region associated with lipids in seedlings’ roots **(A)**, stems **(B)** and leaves **(C)**; and regions related to lipids and pectins in sprouts’ roots **(D)**, stems **(E),** and leaves **(F)**.

**FIGURE 4:**
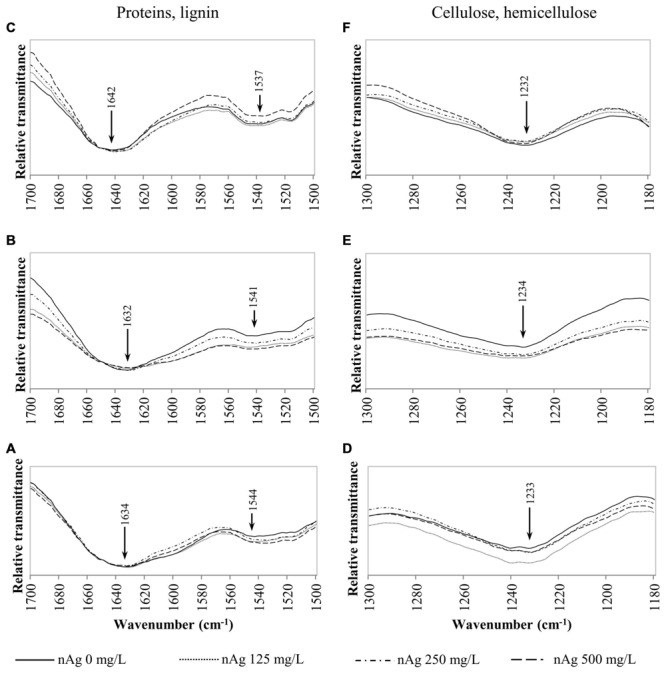
**Overlap ATR-FTIR spectra of radish sprouts exposed to nAg at 0, 125, 250, and 500 mg/L.** Spectral region associated with proteins and lignin in seedlings’ roots **(A)**, stems **(B)**, and leaves **(C)**; and regions related to cellulose and hemicellulose in sprouts’ roots **(D)**, stems **(E)** and leaves **(F)**.

**FIGURE 5:**
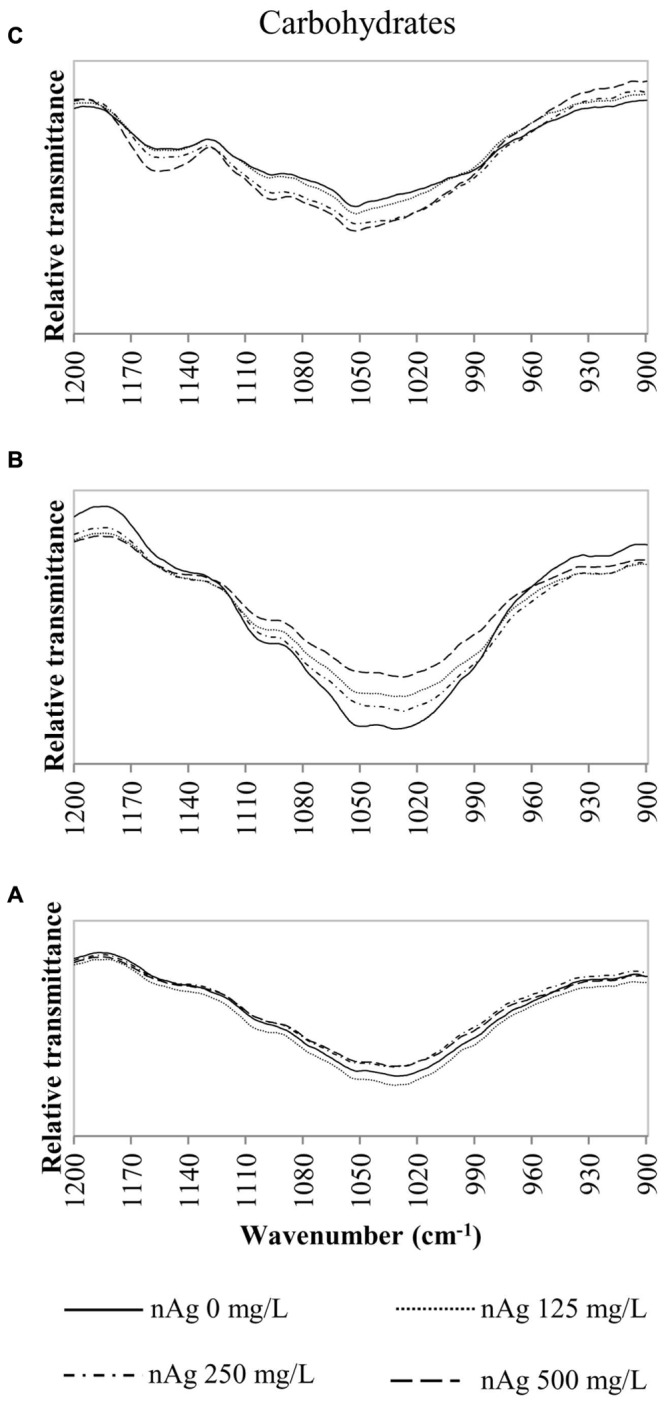
**Overlap ATR-FTIR spectra of radish sprouts exposed to nAg at 0, 125, 250, and 500 mg/L.** Spectral region associated with carbohydrates in seedlings’ roots **(A)**, stems **(B)**, and leaves **(C)**.

Fourier transform infrared spectroscopy has been used to identify conformational changes in the macromolecules of plants exposed to contaminants, including NPs. **Table 2** provides a summary of previously compiled FT-IR data from plant samples that relates the functional groups identified and the macromolecules involved (Dokken and Davis, 2007; Lammers et al., 2009; Rico et al., 2015). For comparison purposes, we chose specific spectral regions where lipids (3000–2800 cm^-1^), proteins and lignin (1700–1500 cm^-1^), lipids and pectin (1790–1720 cm^-1^) cellulose, and hemicellulose (1300–1180 cm^-1^) and other carbohydrates (fingerprint region 1200–900 cm^-1^) are presumably found. Changes in the FT-IR spectra of radish seedlings (**Figures 3–5**) were compared with the data shown in **Table 2**. There are no apparent band shifts in the FT-IR spectra of the roots, stems or leaves treated with nAg at different concentrations as seen in **Figures 3–5**. However, changes in band intensities were found in all studied regions.

**Table 2:** Summary of FT-IR band frequencies found in plants exposed to NPs and other contaminants (Dokken and Davis, 2007; Lammers et al., 2009; Rico et al., 2015).

Frequency (cm^-1^)	Literature freq. (cm^-1^)	Functional group	Molecule/tissue component	From
3100–2800	3100–3000	C–H aromatic	–	Lammers et al., 2009
	3000–2800	C–H aliphatic	–	Lammers et al., 2009
	2960–2940	CH_3_ asymmetric	Lipids	Dokken and Davis, 2007
	2930–2910	CH_2_ asymmetric	Lipids	Dokken and Davis, 2007
	2885–2860	CH_3_ symmetric	Lipids	Dokken and Davis, 2007
	2860–2840	CH_2_ symmetric	Lipids	Dokken and Davis, 2007
1790–1720	1790–1744	C=O	Carboxyl ester, lipids, esterified pectins	Rico et al., 2015
	1749	COOH	Carboxylate COOH, pectin	Lammers et al., 2009
	1742, 1732	C=O	Ester carbonyls, polysaccharides	Lammers et al., 2009
	1740	C=O (alkyl)	Lipids, esterified pectins	Dokken and Davis, 2007
1700–1500	1664–1648	C=O, C–N	Protein	Rico et al., 2015
	1650	C=O, C–N	Protein	Dokken and Davis, 2007
	1635	Aromatic C=C	Lignin	Dokken and Davis, 2007
	1632	Aromatic C=C	Lignin	Rico et al., 2015
	1630–1605	COOH	Carboxylate COOH, pectin	Lammers et al., 2009
	1568–1536	N–H, C–N	Protein	Rico et al., 2015
	1550	N–H, C–N	Protein	Dokken and Davis, 2007
1300–1180	1250–1240	Asymmetric C–O–H	Cellulose, hemicellulose	Dokken and Davis, 2007
	1248–1216	Asymmetric C–O–H	Cellulose, hemicellulose	Rico et al., 2015
1200–900	1200–900	–	Carbohydrate	Dokken and Davis, 2007
	1150–1060	C–O–C (ether)	Lignin	Lammers et al., 2009
	1150–980	C–O	Starch	Lammers et al., 2009
	1130–1050, 1370	-	Cellulose	Lammers et al., 2009
	1072–1040	C–O	Cellulose, hemicellulose	Rico et al., 2015
	1024–992	-	Carbohydrate	Rico et al., 2015
	928–912	-	Carbohydrate	Rico et al., 2015

*Changes in the regions of bands from radish sprouts were compared with data shown in this table*.

## Discussion

### Seed Germination

Nanoparticles tend to agglomerate in suspension due to their size, composition of the medium and ionic strength, among other variables (Murdock et al., 2008). nAg showed moderate agglomeration (77 ± 2.44 nm) and negative surface charge when suspended in Millipore water (Murdock et al., 2008). However, these characteristics did not appear to interact with the cellulosic component of the radish seed coat (Esau, 1977). A previous study has shown that radish is a robust plant, in terms of germination under environmental stresses, due to the hard coat of it seeds that may prevent the entrance of contaminants, like heavy metals and nanoparticles (Koul et al., 2000). Previous studies have also shown no effects of heavy metal solutions or NP suspensions on radish germination. Lane and Martin (1977) reported no penetration of Pb within radish seeds exposed to 95 mg/L of “Analar,” a lead nitrate solution. Lin and Xing (2007) reported that radish germination was not altered by nAl_2_O_3_, nAl, nZn, and nZnO, even at the very high concentration of 2000 mg/L. Wu et al. (2012) found that the EC_50_ for radish germination exposed to nNiO and nCuO was 401 and 398 mg/L, respectively. Trujillo-Reyes et al. (2013) reported no effects on radish seed germination exposed up to 200 mg/L of citric acid coated and uncoated nCeO_2_. Corral-Diaz et al. (2014) reported that germination of radish seeds sown in soil amended with nCeO_2_ at 0*–*500 mg/kg was retarded, but not reduced. Thus, it is not surprising that nAg, even at 500 mg/L, did not affect radish seed germination.

### Silver Uptake, Seedling Growth, Biomass Production, and Water Content

The measurement of heavy metals or NPs’ uptake by plant roots through ICP includes particles/elements adsorbed/ absorbed by the root system (Larue et al., 2012). Hong et al. (2015) showed that washing the tissues with CaCl_2_ and HNO_3_ removed about 80% of the nCeO_2_ sprayed to the leaves of cucumber. Thus, although radish seedlings were washed with HNO_3_ to remove the nAg adhered to the seedlings surface, some particles that could have remained were absorbed by the epidermis. Consequently, the reported data points include both the nAg adsorbed, plus the Ag taken up by the roots. The data reported in the present study, mainly at the highest concentration treatment (500 mg/L), differs from previously reported data. For instance, Song et al. (2013) reported no differences in silver uptake by tomato seedlings developed under exposure to 100 and 1000 mg/L of colloidal silver. These researchers determined that nAg hardly penetrated the hard coat of tomato seeds, and an analogous result could be expected with the hard coat of radish seeds (Koul et al., 2000). In specimens not protected by a hard coat, like rice seeds, penetration of the nAg has been associated with particle size and concentration. Thuesombat et al. (2014) soaked rice seedlings for 24 h in 1000 mg/L of either 20 or 150 nm nAg and germinated the seeds in a sand bed. Seven days after germination, concentrations of Ag in roots were 22 and 12 mg/kg, respectively. In our study, at 500 mg/L the uptake was higher than that reported by Thuesombat et al. (2014) at 1000 mg/L; however, the particles used in the present study were of a smaller size, which could explain the difference. In addition, radish seedlings were in contact with the nAg suspension during the entire experimental period. Results from our study and reports from the literature indicate that the uptake of Ag from nAg depends on a series of factors such as treatment concentration, particle size, plant species, and exposure media.

There is no consistency in the reports of the effects of nAg on seedlings growth. For instance, Lee et al. (2012) reported a reduction of about 60% in mung bean and 75% in sorghum seedlings exposed in agar for 2 days to nAg at 10 mg/L. These reduction rates are considerable larger than those observed in radish, although their support (agar) was different than the suspension used. However, the reduction in radish growth was similar to the reduction in sorghum, but different from that found with mung bean exposed in soil to 800 mg/kg of nAg (Lee et al., 2012), which does not support a comparison between liquid medium or agar. On the other hand, Nair and Chung (2014) exposed rice seedlings for 1 week to nAg in hydroponics (similar conditions of the present study), but at lower concentrations (0.2–1.0 mg/L) and did not report changes in root elongation. This corroborates that the effects of nAg on root growth depend on a series of factors. In addition, differences in treatment concentrations and exposure media made it difficult to compare the results.

Our results on the effects of nAg in biomass production concurs with the reports found in 4-week old rice seedlings exposed to nAg at 0.1–1000 mg/L (Thuesombat et al., 2014). However, they differ from the results with tomato exposed for 15 days to a similar nAg concentration, where the reduction was about 75% (Song et al., 2013). The mechanisms for reduction in biomass production by nAg are not known; however, Nair and Chung (2014) found differential transcription of genes associated with stress tolerance, which could explain the reduction in biomass production.

The reduction in seedlings’ water content under NP exposure has been previously reported. Trujillo-Reyes et al. (2014) found that nCu at 10 and 20 mg/L significantly reduced water content in lettuce. According to the literature, both Cu and Ag block water permeability in roots cells (Niemietz and Tyerman, 2002; Chang et al., 2012), which in turn reduce water absorption. In addition, Qian et al. (2013) proposed that nAg have the potential to change the transcription of antioxidant and aquaporin genes, affecting the balance of water in *Arabidopsis*. It is possible that nAg block aquaporins, reducing the water uptake in radish seedlings. This raises a question which requires more studies for a complete understanding of the aquaporins blockage by nAg.

### Effects of nAg on Macro and Micronutrient Accumulation

Previous studies have shown that the uptake of nutrient elements is affected by both the NM and the species of plant. For instance, Servin et al. (2013) reported increases in Ca, K, and Mg in cucumber exposed to nTiO_2_. Trujillo-Reyes et al. (2014) reported changes on the accumulation of Mn and Zn in lettuce leaves exposed to core-shell Fe/Fe_3_O_4_ and Cu/CuO NPs. Hong et al. (2015) exposed alfalfa and lettuce to several Cu-based NPs/compounds in hydroponics and found changes in the absorption of some macroelements such as K, Mg, and Cu. In addition, Trujillo-Reyes et al. (2013) reported tha CeO_2_ NPs modified the content of Mn and Ni in hydroponically grown radish. In the present study, it was found that nAg reduced the uptake of macroelements Ca and Mg and microelements like B, Mn, and Zn. There is the possibility that nAg decrease the expression of the Ca channel protein, reducing Ca uptake. Magnesium is absorbed in a similar way as bacterial transporters CorA Mg^2+^ (Maathuis, 2009). It is very likely that at high concentrations, nAg are physically blocking the channels, reducing the absorption of Mg.

Boron uptake mechanisms include passive transport through the uncharged boric acid molecule, active transport through boron transporter 1 (BOR1) that uploads B into the xylem, and facilitated diffusion through channels belonging to intrinsic proteins (Dordas et al., 2000; Miwa and Fujiwara, 2010; Wimmer and Eichert, 2013). Trujillo-Reyes et al. (2013) reported that citric acid coated nCeO_2_ reduced the uptake of B in radish; however, to the best of the authors’ knowledge, there is no explanation about the interference of NPs on B uptake. The uptake of Mn and Zn by roots is mediated by putative transporters, Nramp and ZIP family (Guerinot, 2000). The current information is not sufficient enough to get a clear idea of how nAg could affect the uptake of these microelements. As explained above, it is possible that nAg physically block the diffusion pathway or the channels for active absorption. In addition, Magesky and Pelletier (2015) mentioned that silver is a membrane disruptor that breaks down cellular homeostasis. Very likely, this disruption affected the uptake of essential elements. It is also possible that nAg down regulate the genes encoding for metal transporters. Further investigation is needed in order to unravel the mechanism of nAg interference with the uptake of micronutrients.

### FT-IR Functional Groups/Macromolecules Analysis

According to **Table 2**, the C–H bond in CH_2_–CH_3_ groups associated with lipids is found in the region of 3000–2800 cm^-1^. These macromolecules are constituents of the lipid bilayer found in cell membranes. **Figures 3A–C** shows the relative transmittance of bands in the spectra obtained for roots, stems and leaves assigned to lipids in radish sprouts. Bands in the region between 1790 and 1720 cm^-1^ (**Figures 3D–F**) are associated to C=O and COOH groups assigned also to lipids and pectins, these last are made of polysaccharides and are accountable for the structure of the primary cell wall.

**Figures 4A–C** shows the peaks found at 1650–1630 cm^-1^ for lignin and bonds identified for proteins at 1650 cm^-1^, 1549–1530 cm^-1^ corresponding to C=O, N–H and C–N (Dokken and Davis, 2007; Rico et al., 2015). Changes in cellulose and hemicellulose are observed at 1242–1230 and 1054–1051 cm^-1^ in **Figures 4D–F** and **5** respectively. Also, carbohydrates not attributed to a specific biopolymer are shown from 1200–900 cm^-1^ in **Figure 5**.

Alterations in lipids and carbohydrates were similar to those reported for cilantro exposed to nCeO_2_ (Morales et al., 2013). While hemicellulose, cellulose and pectin are structural components of primary cell walls, lignin provides rigidity to terrestrial plants (Cabane et al., 2012). Disruption in these macromolecules may lead to changes in morphology that could impair the normal development of the plants. The fact that no band shifts were observed suggests that there are no chemical changes in the macromolecules studied, only shape alterations in the plant tissue components (Morales et al., 2013).

In summary, concentrations of nAg used in this study did not affect radish seed germination. However, there was a concentration-dependent reduction in seedling elongation and water content. In addition, at 250 mg/L the biomass was reduced by 10%, compared with control (*p* ≤ 0.05). Silver NPs also impaired the absorption of nutritional elements in radish seedlings. Important macroelements such as Ca and Mg and microelements B, Cu, Mn, and Zn were reduced by the highest concentration of nAg. Moreover, nAg induced conformational changes in carbohydrates, lignin, and lipids. The impacts of such changes in the nutritional value of radish sprouts are not known yet. In addition, as per Holden et al. (2014), “it may be premature for manufactured NMs risk research to sanction information on the basis of concentration ‘environmental relevance’.”

## Author Contributions

All authors listed, have made substantial, direct and intellectual contribution to the work, and approved it for publication.

## Conflict of Interest Statement

The authors declare that the research was conducted in the absence of any commercial or financial relationships that could be construed as a potential conflict of interest.

## Footnotes

1 http://www.nanotechproject.org/cpi/

2 http://www.npswsilver.com/
