# Supplementary material for: Effects of Silver Nanoparticles on Radish Sprouts: Root Growth Reduction and Modifications in the Nutritional Value
Source: Front Plant Sci. 2016 Feb 16;7:90. doi: 10.3389/fpls.2016.00090 (PMC4754487; doi:10.3389/fpls.2016.00090)
Supplement: Supplementary file 1 [file Data_Sheet_1.DOCX]

Control,

nAg 0 mg/L

nAg 125 mg/L

nAg 250 mg/L

nAg 500 mg/L


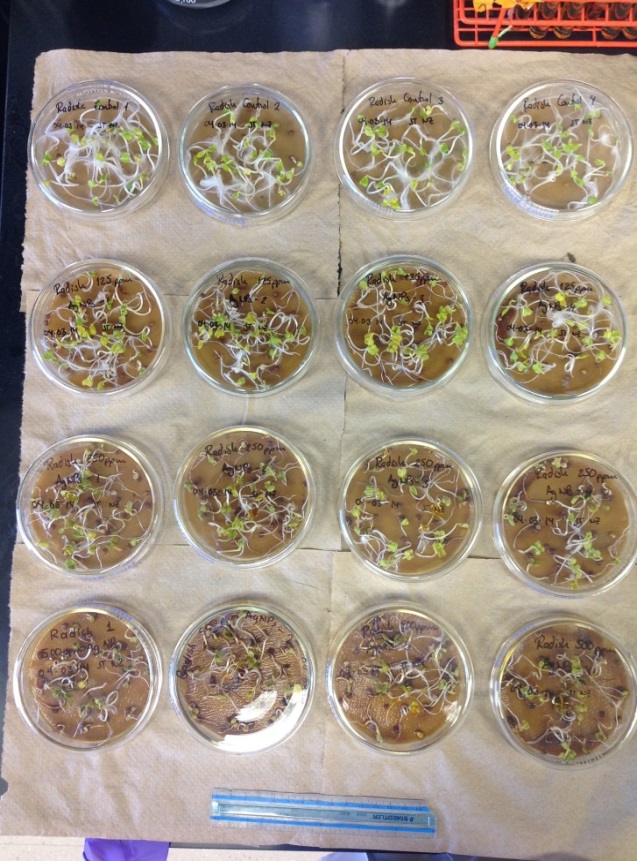


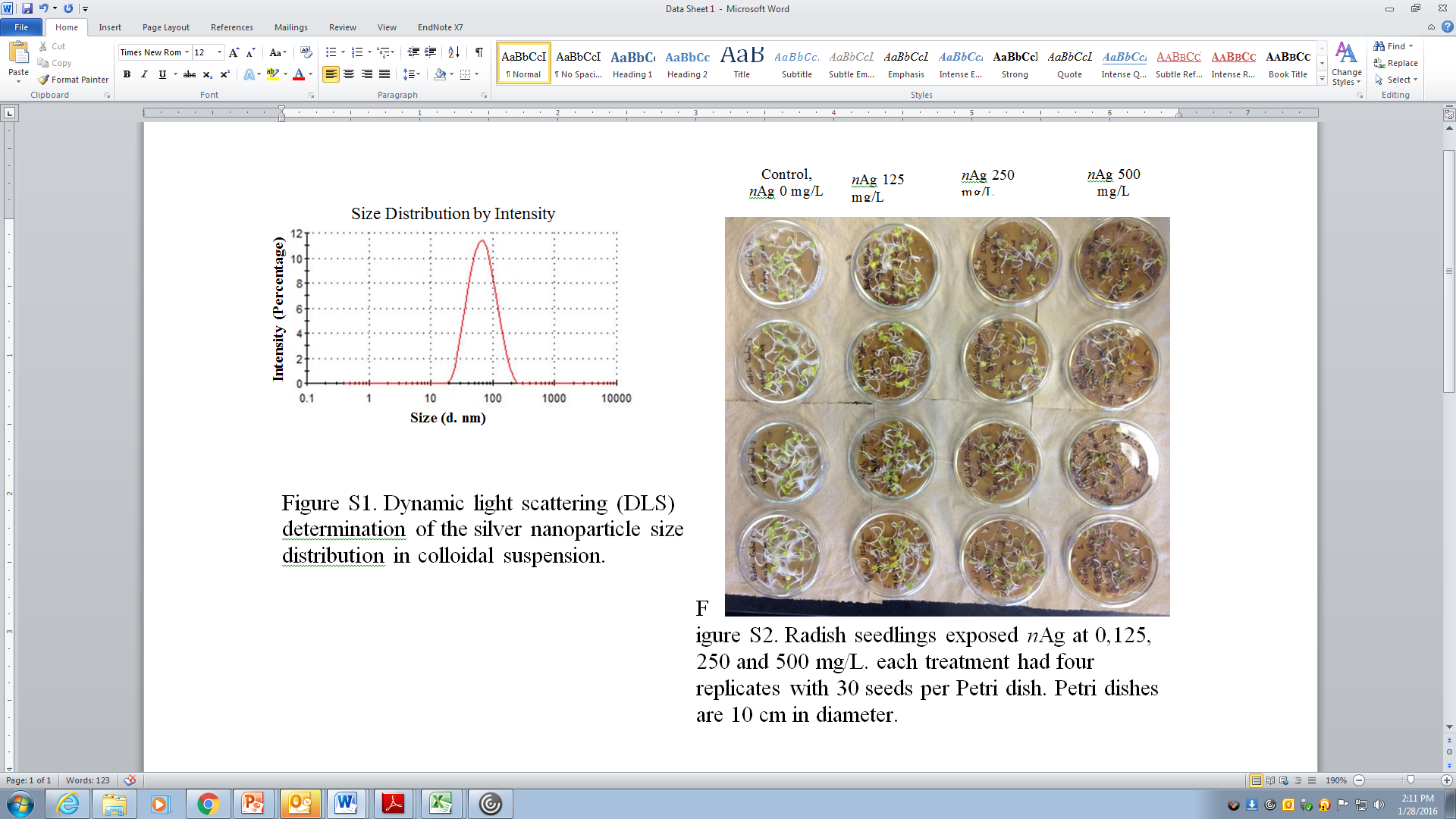


**Figure S1**. Dynamic light scattering (DLS) determination of the silver nanoparticles’ size distribution in colloidal suspension.


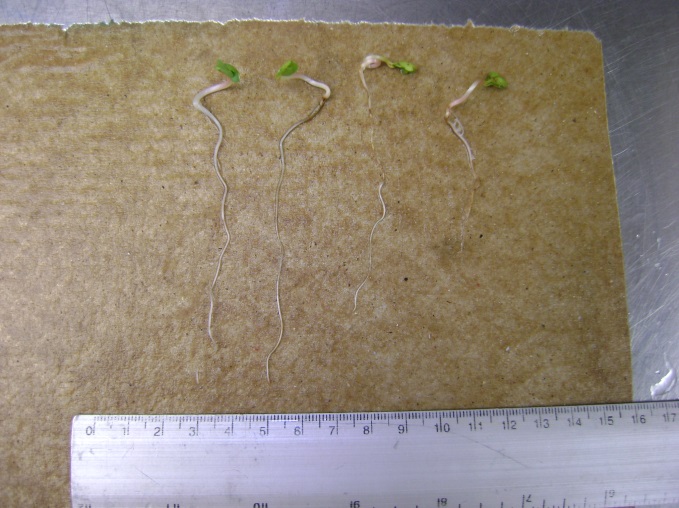


Control,

nAg 0 mg/L

nAg 125 mg/L

nAg 250 mg/L

nAg 500 mg/L

**Figure S4**. Radish sprouts unraveled from petri dish. Image provides a visual perception of the effects that different nAg concentrations have on radish seedlings growth.

**Figure S3**. Five day-old radish seedlings exposed to nAg at 0, 125, 250 and 500 mg/L.


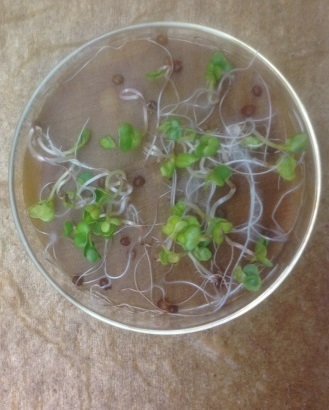


nAg 125 mg/L


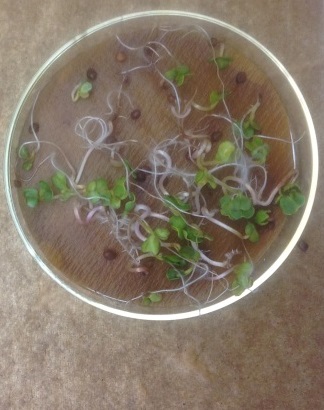


nAg 250 mg/L


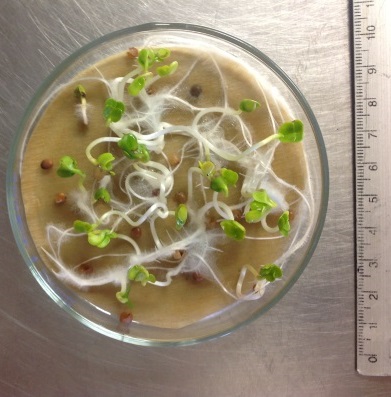


Control, nAg 0 mg/L


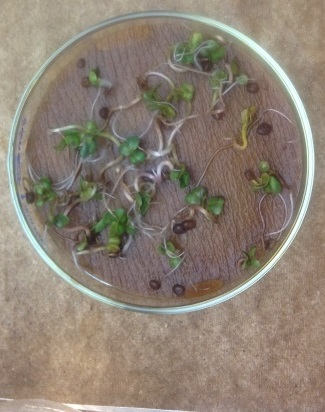


nAg 500 mg/L

**Figure S2**. Experimental setup: radish seedlings exposed nAg at 0, 125, 250 and 500 mg/L. Each treatment had four replicates with 30 seeds per Petri dish. Petri dishes are 10 cm in diameter.
